# flexiMAP: a regression-based method for discovering differential alternative polyadenylation events in standard RNA-seq data

**DOI:** 10.1093/bioinformatics/btaa854

**Published:** 2020-11-25

**Authors:** Krzysztof J Szkop, David S Moss, Irene Nobeli

**Affiliations:** Department of Biological Sciences, Institute of Structural and Molecular Biology, Birkbeck, University of London, London WC1E 7HX, UK; Science for Life Laboratory, Department of Oncology‐Pathology, Karolinska Institutet, Solna, Sweden; Department of Biological Sciences, Institute of Structural and Molecular Biology, Birkbeck, University of London, London WC1E 7HX, UK; Department of Biological Sciences, Institute of Structural and Molecular Biology, Birkbeck, University of London, London WC1E 7HX, UK

## Abstract

**Motivation:**

We present flexible Modeling of Alternative PolyAdenylation (flexiMAP), a new beta-regression-based method implemented in *R*, for discovering differential alternative polyadenylation events in standard RNA-seq data.

**Results:**

We show, using both simulated and real data, that flexiMAP exhibits a good balance between specificity and sensitivity and compares favourably to existing methods, especially at low fold changes. In addition, the tests on simulated data reveal some hitherto unrecognized caveats of existing methods. Importantly, flexiMAP allows modeling of multiple known covariates that often confound the results of RNA-seq data analysis.

**Availability and implementation:**

The flexiMAP *R* package is available at: https://github.com/kszkop/flexiMAP. Scripts and data to reproduce the analysis in this paper are available at: https://doi.org/10.5281/zenodo.3689788.

**Supplementary information:**

Supplementary data are available at *Bioinformatics* online.

## 1 Introduction

Alternative polyadenylation (APA) is the selection of alternative cleavage and polyadenylation sites during transcription of eukaryotic genes, resulting in isoforms with distinct lengths. APA has been shown to be prevalent in mammalian transcripts and alternative isoforms are linked to different stages of development, cell types and disease status (Elkon *et al*., 2013; Szkop *et al*., 2017). APA events can be identified on a genome-wide scale using 3′ end-focused sequencing [e.g. QuantSeq (Moll *et al*., 2014)] or, more recently, long-read sequencing [Iso-seq (Anvar *et al*., 2018) and nanopore-based sequencing (Garalde *et al*., 2018)]. However, as these methods are still not widely used and many legacy transcriptome surveys were carried out using standard RNA-seq sequencing instead, it would be useful to have computational methods that can identify differential APA in RNA-seq data. A few such methods exist already (Xia *et al*., 2014; Grassi *et al*., 2016; Ha *et al*., 2018; Ye *et al*., 2018; Arefeen *et al*., 2018) but they have caveats (Szkop and Nobeli, 2017). For example, all methods must solve the problem of how to deal with biological replicates; some test the replicates individually, losing the advantage of having replicates in the first place; others, average values from replicates, effectively losing track of the within-group variability. In designing a method for differential APA analysis, we considered the following: (i) the reconstruction and quantification of the individual isoforms is both challenging and not strictly necessary for this task; (ii) the errors in modeling RNA-seq read counts are neither normal nor Poisson-distributed; (iii) multiple covariates can affect APA.

Inspired by the use of Generalized Linear Models (GLMs) in differential gene expression (Robinson *et al*., 2010; Love *et al*., 2014) we present here a regression-based method and associated pipeline (flexible modeling of APA or flexiMAP) that satisfactorily addresses the above requirements. We show, using simulated data, that the method is both sensitive and specific across a range of fold changes and numbers of samples and that its performance is superior to two alternatives [DaPars (Xia *et al.*, 2014), and APAtrap (Ye *et al.*, 2018)] in most tests we carried out. FlexiMAP is also outperforming both these methods and Roar (Grassi *et al.*, 2016), when additional covariates confound changes to the isoform ratios. Tested on real RNA-seq data, flexiMAP is slightly less specific than the other methods tested but outperforms all methods when the Matthews Correlation Coefficient is used as the measure of performance, indicating a better overall balance between specificity and sensitivity.

The method is available as an *R* package from: https://github.com/kszkop/flexiMAP

## 2 Materials and methods

Our method can be applied to all pairs of polyadenylation sites in a gene, where one site is considered ‘distal’ (i.e. located furthest away from the end of the coding region) and one is ‘proximal’ (Supplementary Fig. S1). Given a list of sites provided to the program, pairs of sites will be considered in turn, the most downstream site of the transcript being the distal site in all pairs. The proximal site separates the 3′ UTR into two regions: the ‘short’ region, starting at the end of the coding region and ending at the proximal site, and the ‘long’ region starting at the proximal site and ending at the end of the transcript (Supplementary Fig. S1). Assuming the separation of samples into groups based on the condition of interest, the question we want to answer is: given a total number of reads falling in the 3′ UTR, is the proportion of reads falling in the long region dependent on the sample group membership?

We count RNA-seq reads falling in the ‘long’ and ‘short’ regions of the 3′ UTR (*N*_long_^*ij*^ and *N*_short_^*ij*^, respectively), and define the ratio, *R*, for gene *i* in sample *j* as:

(1) $$R_{ij}=\frac{N_{{\text{long}}^{ij}}}{{N_{{\text{short}}^{ij}}+N}_{{\text{long}}^{ij}}}$$

Reads falling in the long region can only originate from transcripts using the distal site, whereas reads falling in the short region may come from transcripts using either the distal or the proximal site. The ratio *R_ij_* is the proportion of reads falling in the long region and is thus strictly contained in the interval [0,1]. We note that the extreme value of zero is only encountered in the complete absence of a long isoform, whereas values greater than 0.5 would be observed only in cases where the long region is longer than the short region, or where strong 3′ biases in the read coverage are observed.

Our initial tests modeling APA events using logistic regression with quasi-binomial error distribution (within the GLM framework) showed that this approach was not sensitive enough for small numbers of samples or small fold changes. To allow more flexibility in modeling errors, we adopted instead a model where the response variable is assumed to be beta-distributed. This beta-regression model was implemented using the *betareg* package in *R* (Cribari-Neto *et al*., 2010). In addition, the quasi-binomial GLM is implemented in our software and used for transcripts where the number of reads falling in the long region is zero, as the ratio in these cases falls outside the permitted values for modelling with beta regression.

Finally, our method incorporates two filtering steps to improve accuracy, employing TIN (Transcript Integrity Number) values (Wang *et al*., 2012, 2016) to filter on transcript integrity and removing transcripts with too few reads mapping to the short region (see Supplementary Methods for details).

## 3 Results

**Fig. 1.**
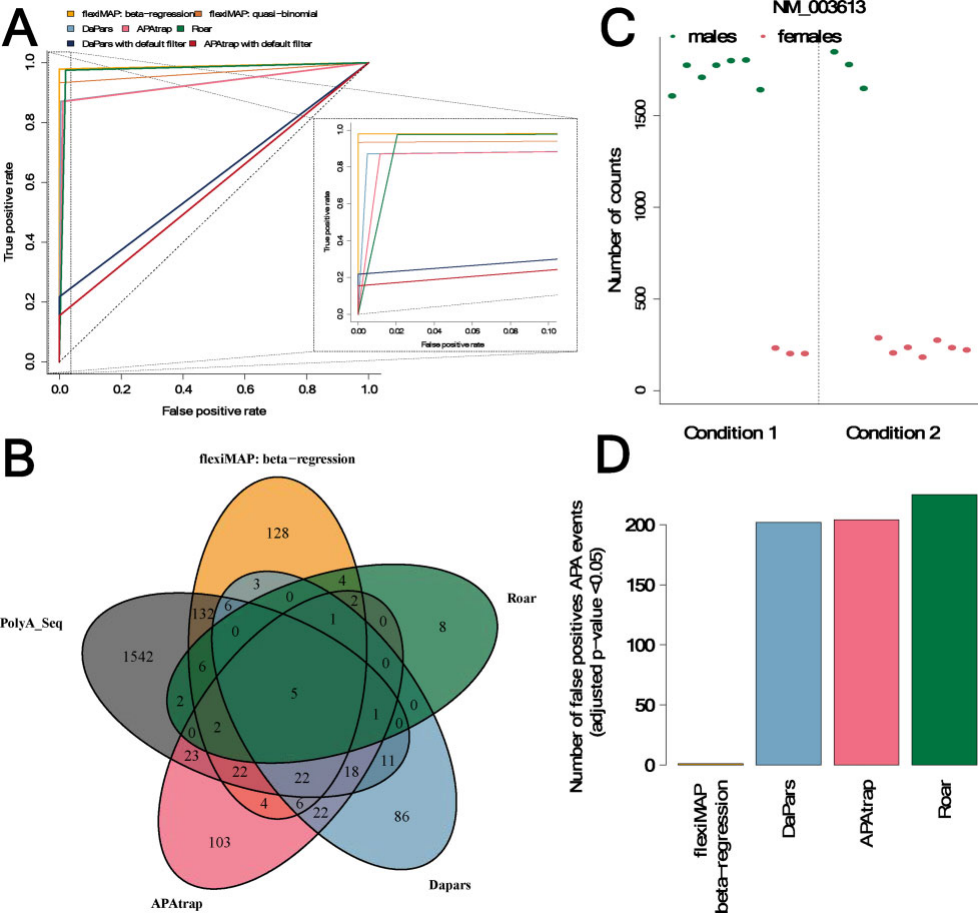
flexiMAP detects differential polyadenylation events with a good balance of specificity and sensitivity. (**A**) Receiver operating characteristic (ROC) curves representing the accuracy of detecting differential APA events using flexiMAP, DaPars, APAtrap and Roar. DaPars and APAtrap make their own prediction of polyadenylation sites, not always agreeing with the annotated sites used in this study. To avoid inflating the error rate of these programs by including sites that do not map the annotation (and hence, differential events called at these sites would be automatically considered as false positives), only transcripts where the polyadenylation site was correctly predicted by DaPars and APAtrap are included in this plot. FlexiMAP clearly outperformed DaPars, APAtrap and Roar by perfect specificity and improved sensitivity in this simulated experiment. Although application of the DaPars’ PDUI (Percentage of Distal polyA site Usage Index) *post hoc* filter (dark blue) and APAtrap’s PD (Percentage Difference) filter (dark red) corrected the false positives problem of these methods, they did so at a heavy cost on sensitivity. (**B**) Venn diagram showing the overlap of ‘true’ differential polyadenylation events in the MAQC samples PolyA-seq data (as called by DEXSeq; grey) with predictions from all four methods tested here: flexiMAP (orange), DaPars (light blue), APAtrap (pink) and Roar (green). (**C**) Example from the imbalanced simulated dataset of a situation where a covariate of no interest (in this case, sex) affects the ratio of reads assigned to short and long isoforms. Male samples display much higher expression of the short region of transcript NM_003613 compared with female ones, regardless of the condition group samples belong to. In addition, the dataset is imbalanced, with more males present in condition 1 than condition 2. The mean expression for condition 1 is thus higher than the mean for condition 2, but the effect is due to the covariate sex, not the condition to which the samples belong to. (**D**) DaPars, APAtrap and Roar report a large number of false positives for an imbalanced simulated dataset. In contrast, flexiMAP reports only one false positive in this case, highlighting its main advantage over alternative approaches

We compared flexiMAP to three existing methods for APA analysis [DaPars (Xia *et al.*, 2014), Roar (Grassi *et al.*, 2016) and APAtrap (Ye *et al.*, 2018)] using simulated data we produced with the *polyester* R package (Frazee *et al*., 2015) (see Supplementary Methods for details). In these tests, our method is specific (none of the transcripts with no APA events are predicted as having such events) and outperforms in sensitivity DaPars and APAtrap up to a fold change of four (Fig. 1A, Supplementary Fig. S2). For larger fold changes, all methods appear to perform equally well. Surprisingly, the application of post-detection filters recommended by the developers of both DaPars and APAtrap appear to remove the majority of significant events across all fold changes, which renders questionable the usefulness of these filters (Supplementary Fig. S2). In these simulations, Roar is more sensitive than flexiMAP at small fold changes but it is also the least specific, having the largest number of false positives of all methods compared. We note that the performance of Roar is dependent on the parameter value that controls the filtering of significant events (nUnderCutoff; set here to 50%) and that the specificity of the method can be improved by increasing this parameter, albeit at a great cost in sensitivity at low fold changes (Supplementary Fig. S2).

All methods, including flexiMAP, were sensitive to the expression level of the transcript tested for differential polyadenylation (Supplementary Fig. S3). APA events that were missed originated in transcripts of lower overall expression but the beta-regression approach displayed improved sensitivity over all of the other methods, except Roar. Unlike methods that average across samples from the same condition, the performance of flexiMAP depends on the number of samples available in each group, as expected for a method that needs to model the variance within each group (see Supplementary Fig. S4). However, flexiMAP is much more sensitive than the GLM-quasi-binomial method at small sample sizes (<6), often encountered in RNA-seq datasets. Finally, flexiMAP’s sensitivity does not seem to be affected by the length of the 3′ UTR (Supplementary Fig. S5).

Although simulated datasets are important for benchmarking tests, eventually methods are only useful if they can be applied to real data. The dataset we used here is the same used by both DaPars and APAtrap in their respective publications and contains RNA-seq data from the Human Brain Reference and the Universal Human Reference MAQC samples (Bullard *et al*., 2010). 3′ sequencing data (PolyA-seq) for the same samples was downloaded from the UCSC genome browser [processed with an independent method, DEXSeq (Anders *et al*., 2012), to call the ‘true’ differential polyadenylation events, as described in Supplementary Methods]. The results of applying all methods to this dataset (Fig. 1B) demonstrate that all four miss a large number of events called by DEXseq but flexiMAP is the most sensitive method as well as the one with the highest Matthews Correlation Coefficient [MCC; 0.27 for flexiMAP as compared with 0.23 (Roar), 0.15 (APAtrap) and 0.1 (DaPars)]. FlexiMAP’s specificity is lower in this dataset compared with other methods but remains over 0.9. Given these results, we believe that although filters or more conservative cut-offs for significance could reduce the number of false positive events called by flexiMAP, they may only be useful in practice when very high specificity is required.

The development of flexiMAP was primarily driven by the need to model multiple known covariates in APA analysis. Indeed, flexiMAP successfully discriminates between the effect of the condition of interest and that of an additional covariate in a simple simulated scenario of imbalanced datasets, where APA originates from the sex attribute of the samples rather than the condition of interest (Fig. 1C and D). Similar results are obtained with a more complex simulated dataset with two covariates (see description in Supplementary Methods and results in Supplementary Fig. S6). Clearly, this is still an artificially simple scenario and one would expect more false positives in real data where at least some of the batch effects might be unknown and hence not included in the modelling. In addition, many real RNA-seq datasets still do not have enough samples to allow successful modelling of multiple covariates so flexiMAP’s accuracy as measured in these simulations is likely to be lower with real data. However, it is clear that methods that are not designed to take into account multiple covariates will naturally misinterpret the origin of the variation, resulting in increased false positive rates.

## 4 Conclusion

We presented here flexiMAP, a beta-regression-based method for detecting APA events in RNA-seq data, given a list of putative polyadenylation sites. Our method is both sensitive and specific, even when small numbers of samples are used, and has the distinct advantage of being able to model contributions from known covariates that would otherwise confound the results of APA analysis. FlexiMAP compares favourably with existing alternatives in tests involving simulated datasets. Importantly, these tests have highlighted some hitherto overlooked caveats of existing methods. Real datasets remain a challenge for all methods, not least because it is difficult to define objectively the ground truth, but flexiMAP is still outperforming other methods, when both specificity and sensitivity are taken into account using the Matthews Correlation Coefficient.

## Supplementary Material

btaa854_Supplementary_Data
