# Supplementary material for: flexiMAP: a regression-based method for discovering differential alternative polyadenylation events in standard RNA-seq data
Source: Bioinformatics. 2020 Nov 25;37(10):1461–4. doi: 10.1093/bioinformatics/btaa854 (PMC8208744; doi:10.1093/bioinformatics/btaa854)
Supplement: btaa854_Supplementary_Data [file btaa854_supplementary_data.docx]

**SUPPLEMENTARY INFORMATION**

**flexiMAP: A regression-based method for discovering differential alternative polyadenylation events in standard RNA-seq data**

Krzysztof J. Szkop, David S. Moss and Irene Nobeli

# Supplementary Methods

## Description of the flexiMAP method

Given a catalogue of polyadenylation sites for a genome, the most downstream site per gene is selected as the end of the transcript and all other sites that are downstream from the coding region of a gene are potential alternative (proximal) polyadenylation sites. Each proximal site defines two regions in the 3’ UTR (Supp. Fig. 1). The term “short” region is used here to refer to the part of the transcript starting from the start of the 3’UTR and ending at the proximal polyadenylation site and the term “long” region to refer to the part starting from the proximal site and ending at the most downstream polyadenylation site. It is important to emphasize that multiple proximal sites in one transcript are possible. All known sites belonging to one transcript are included in the flexiMAP analysis, but each proximal site is modeled separately.

Using the raw number of RNA-seq reads falling on each of these regions, the ratio *R* is calculated for each transcript (*i*) in each sample (*j*) using:

$R_{ij}=\frac{N_{\text{long}^{ij}}}{{N_{\text{short}^{ij}}+N}_{\text{long}^{ij}}}$, (1)

where *N_long_^ij^* and *N_short_^ij^* are the number of reads falling in regions *long* and *short* respectively of transcript *i* in sample *j*. It is worth pointing out here that reads falling in the long region can only originate from transcripts using the distal site, whereas reads falling in the short region may come from transcripts using either the distal or the proximal site. The ratio *R* has the desired property of being nearly always constrained in the interval (0,1); the extreme value 0 is only possible in the complete absence of the long isoform and values greater than 0.5 would generally only be observed if the long region was longer than the short region or if the dataset was affected by 3’ or other biases in the distribution of reads (both of which could lead to a much larger number of reads covering the long region, compared with the short region). Hence, limiting modelling of *R* to the interval (0,1) is acceptable in most cases.

Response variables representing proportions, like the ratio *R* above, are commonly modelled using logistic regression. A link function (e.g. “logit”) relates the mean of the response variable, *y*, to a linear predictor, *η*, and errors are usually assumed to be binomial. Count data in real experiments is often over-dispersed and in this case, the quasi-binomial family of errors can be used instead, thus doing away with the requirement of knowing in advance the relationship between mean and variance. In the context of APA, the main explanatory variable of interest is likely to be categorical (a“condition”), resulting in an ANOVA-like analysis of deviance.

In our own trials, modelling APA events using logistic regression with quasi-binomial error distribution (within the Generalised Linear Model framework in R) had poor sensitivity, when the number of samples was small or when small fold changes were involved. This suggested that perhaps the error distribution needed to be modeled more flexibly. Hence, we decided to adopt a model where the response variable is beta-distributed. The formula for the beta density, as parameterised by (Ferrari and Cribari-Neto, 2004) is given by the following equation:

$f\left( y;\mu,\phi\right)=\frac{\Gamma(\phi)}{\Gamma(\mu\phi)\Gamma(\left( 1-\mu\right)\phi)}y^{\mu\phi-1}{(1-y)}^{\left( 1-\mu\right)\phi-1}$ , (2)

where 0 < *μ* < 1 is the mean of *y* and *φ* is known as the precision parameter, with *φ* > 0. Γ denotes the gamma function. The variance of *y* is given by *μ*(1-*μ*)/(1+*φ*), with precision parameter, *φ*, allowing for wide range of shapes for the density. The beta distribution‘s density can have very different shapes depending of the value of the two parameters that determine the distribution, offering flexibility for modeling proportions. Furthermore, the interpretation of the results of a beta regression is similar to that of logistic regression with quasi-binomial errors. A beta-regression model was implemented here using the *betareg* package in R (Cribari-Neto and Zeileis, 2010). We experimented with different link functions (Supp. Fig. 7) but results were not very sensitive to the selection of the link function in this case (we have chosen to use “loglog” in our manuscript). The package allows an extension to the original beta-regression model, where the precision parameter, *φ*, is not assumed constant between observations but it is instead modeled in a similar fashion to the mean. However, we have not tested the use of a variable precision parameter as we believe the limited amount of data available would be problematic for modeling both parameters.

Our method currently relies on knowledge of the polyadenylation sites, usually obtainable from public databases. In a future version, the option of predicting sites from the density of RNA-seq reads could be added as a pre-processing step. However, this is not trivial. Even in simulated data, where read coverage of the 3’UTRs is perfect, DaPars predictions of proximal sites fall outside a 50-nucleotide window tolerance for around 40% of the sites (Supp. Fig. 8B).

Our method incorporates two additional steps to improve accuracy. The first addresses the issue of distorted results in cases of severe RNA degradation levels. TIN values are pre-calculated as described in RSeQC (Wang *et al.*, 2012) to measure RNA integrity at transcript level and transcripts below a TIN threshold are removed. The second step addresses the problem of low expression by removing transcripts with fewer than a predefined threshold number of reads (20) mapping to the “short” region.

A flow diagram of the flexiMAP method is shown in Supp. Fig. 9.

We note that adapting flexiMAP to be used for the analysis of data from long-read sequencing is relatively straightforward; relevant changes to the code are planned for future releases.

## Details of simulated data

An “idealized” dataset of RNA-seq reads was created using the *polyester* R package (Frazee *et al.*, 2015). This simulated dataset is clean of technical biases and fold changes between isoforms are known, allowing testing of the sensitivity limits of the method in the absence of external factors. The simulation experiment comprises 20 samples, 10 in each of two conditions. Polyadenylation sites splitting each transcript into two isoforms (short and long) were obtained from the poly(A) site atlas (Gruber *et al.*, 2016) for 11000 human transcripts. Each isoform (“short” and “short + long”) was simulated as a different transcript. The expression of the “shot + long” isoform was unchanged between conditions, whereas eleven different fold changes were applied between conditions for the “short” isoform in order to produce a range of different ratios, *R*. Hence, each fold change is represented by ~ 1000 transcripts in the dataset. Additionally, for each fold change category we assigned 100 different mean expression levels (from 100 to 1000) with the aim of sampling the effect of the expression level on the ability of the method to detect alternative polyadenylation events.

In order to assess the performance of flexiMAP in dealing with multiple covariates, two additional small datasets were simulated. These datasets comprised 1000 and 3000 transcripts respectively (instead of the original 11000) to speed up the calculation time taken by the slowest method tested here, APAtrap. The aim was to create a scenario where fold changes between two conditions are confounded by the presence of additional factors. The first dataset contains a single covariate implemented by an imbalanced setup where male and female-origin samples are present in unequal numbers in the control (7 males and 3 females) and condition (3 males and 7 females) groups. Although the group membership for the factor of interest (condition) plays no role in the choice of polyadenylation site of these transcripts, membership to male or female group does, confounding the outcome of methods that do not take into account additional covariates. The second dataset is more complex with two confounding factors (age and sex). The control group consists of 5 young and 2 old males and 1 old and 2 young females. The second group consists of 1 young and 1 old male and 4 young and 4 old females. Similarly to the first example, group membership plays no role in the choice of polyadenylation site and no significant events should be called by any method.

## Details of real data

The real data used to test methods in this study is the same used in the original publications of DaPars and APAtrap. This RNA-seq dataset was obtained by sequencing samples from the MAQC consortium: one group of samples corresponds to the Human Brain Reference and the second corresponds to the Universal Human Reference MAQC samples (Bullard *et al.*, 2010). As real data often contains degraded transcripts, TIN values were calculated using RSeqC to estimate transcript integrity. Transcripts with TIN values less than 40 were removed. Transcripts were also filtered out if there were fewer than 20 reads mapping to the “short” region, as we argued that in this case there would not be enough data to come to a conclusion about differential polyadenylation events. The “ground truth” for significant events was taken to be the PolyA-seq data available for a subset (4) of these samples, downloaded from the UCSC human genome browser ((<http://genome.ucsc.edu/>). Statistically significant changes in poly(A) site use between conditions were identified using DEXSeq (Anders *et al.*, 2017). We note that DaPars and APAtrap both used a different approach to identify significant events in the PolyA-seq data but in each case essentially the same method was used as was applied later to the RNA-seq dataset. We believe this is biasing the results in favour of the method used on the PolyA-seq events. Hence, we opted to use instead an independent method (DEXseq) for calling significant events in the 3’ data.

All other processing and analysis of real data followed the same pipeline as that used for the simulated data in this study as outlined in the sections below.

## Quality control assessment of simulated data

A simulated dataset was used to assess the performance of flexiMAP. As differences in the length of the 3’ UTRs across groups of transcripts could bias the results, we first checked the distribution profiles of the 3’UTR lengths across all the simulated groups (Supp. Fig. 8A). Following filtering of transcripts with incorrectly predicted proximal polyadenylation sites by DaPars and APAtrap, on average, approximately 43% of transcripts were kept in each fold change group.

## Mapping and counting reads in simulated data

Reads from the simulated dataset were mapped to the hg19 reference genome using HISAT2(Kim *et al.*, 2015). Uniquely mapped reads to non-overlapping genomic features were used as input to flexiMAP. Reads were counted using the *featureCounts* function from the Bioconductor package *Rsubread* (Liao *et al.*, 2014).

## Assessing differential polyadenylation with DaPars

Once reads were mapped, the resulting SAM-formatted files were converted to the *bedgraph* format using Bedtools v2.17.0 (Quinlan and Hall, 2010) and these were used as input for the software DaPars-v.0.9 (Xia *et al.*, 2014). DaPars discovers statistically significant alternative polyadenylation events between two groups of samples. It predicts proximal sites using the drop in the number of reads near the site as a signal in a two-point model. In the case of simulated data, the proximal polyadenylation sites are already known and there is no need to predict them. Hence, in order to facilitate comparison between DaPars and other methods, we considered only cases where proximal polyadenylation sites were correctly predicted by DaPars (a correct prediction being defined here as a site that is located within 25 nucleotides in either direction of the known proximal site). Differences in the use of polyadenylation sites between treated and untreated samples were examined using the PDUI value, as defined by the software DaPars. The PDUI value is calculated for each gene passing the coverage thresholds of DaPars and is a measure of the preference of using the distal over the proximal poly(A) site (the DaPars software assumes and compares only two sites). If all reads are assigned to the distal site, the PDUI value is 1, and if all reads are assigned to the proximal site, 0. Mean PDUI values are calculated within each group and contrasted between groups to discover significant differential alternative polyadenylation events.

## Assessing differential polyadenylation with APAtrap

A full analysis of the simulated dataset using APAtrap is estimated to take approximately 6 months on our server (based on the estimator built into the software), so it was decided to apply this method only to the subset of transcripts with proximal polyadenylation sites correctly predicted by DaPars. The “Percentage Difference” (PD) is calculated by APAtrap to quantify the difference of APA site usage in a given gene between samples. A linear trend test based on the Pearson product moment correlation coefficient is employed to check the trend in proportions of APA usage and the false discovery rate is calculated using the Benjamini-Hochberg method.

## Assessing differential polyadenylation with Roar

The method was applied to mapped simulated reads. The Roar ratio was calculated to quantify the difference of APA site usage in a given gene between samples. The Roar method assigns significance to the differences in APA site usage by carrying out pairwise comparison of samples between conditions using a Fisher exact test. The number of significant *p*-values for all pairwise comparisons is then counted and a minimum number is set as a cut-off for removing insignificant events. The Roar developer used (in the package documentation) a value of 100% as the filter, a conservative value meaning that all pairwise tests must be significant. When we applied this filter to the real dataset used here, Roar failed to identify any significant events. After trials, we decided to set this filter to 50% as we believe this results in a reasonable balance between specificity and sensitivity for both real and simulated datasets.

## Availability

The flexiMAP *R* package is available from:

<https://github.com/kszkop/flexiMAP>

Scripts and data to reproduce the analysis in this paper have been uploaded at the Zenodo repository (<https://doi.org/10.5281/zenodo.3689788>).

## References

Anders, S. *et al.* (2012). Detecting differential usage of exons from RNA-seq data. Genome Research, **22**, 4025.

Bullard, J.H. *et al.* (2010). Evaluation of statistical methods for normalization and differential expression in mRNA-Seq experiments. *BMC Bioinformatics*, **11**, 94.

Cribari-Neto,F. and Zeileis,A. (2010) Beta Regression in R. *J. Stat. Soft.*, **34**, 1–24.

Ferrari,S.L. and Cribari-Neto,F. (2004) Beta regression for modelling rates and proportions. *Journal of Applied Statistics*, **31**, 799–815.

Frazee,A.C. *et al.* (2015) Polyester: simulating RNA-seq datasets with differential transcript expression. *Bioinformatics*, **31**, 2778–2784.

Gruber,A.J. *et al.* (2016) A comprehensive analysis of 3' end sequencing data sets reveals novel polyadenylation signals and the repressive role of heterogeneous ribonucleoprotein C on cleavage and polyadenylation. *Genome Res.*, **26**, 1145–1159.

Kim,D. *et al.* (2015) HISAT: a fast spliced aligner with low memory requirements. *Nat. Methods*, **12**, 357–360.

Liao,Y. *et al.* (2014) featureCounts: an efficient general purpose program for assigning sequence reads to genomic features. *Bioinformatics*, **30**, 923–930.

Quinlan,A.R. and Hall,I.M. (2010) BEDTools: a flexible suite of utilities for comparing genomic features. *Bioinformatics*, **26**, 841–842.

Xia,Z. *et al.* (2014) Dynamic analyses of alternative polyadenylation from RNA-seq reveal a 3'-UTR landscape across seven tumour types. *Nat Commun*, **5**, 5274.

# Supplementary Figure 1

**Definition of “short” and “long” regions in the 3’ UTR of transcripts**

An alternative (proximal) poly(A) site in the 3’ untranslated region of a eukaryotic transcript splits this UTR into two parts, the “short” and the “long” regions. Isoforms terminating at the proximal site will only contain the short region whereas isoforms terminating at the distal site (often considered to be the canonical site) will contain both short and long regions. In this diagram, the coding part of the exon is shown as a thick rectangle whereas the 3’ UTR is shown as the thinner rectangle.

# Supplementary Figure 2

**flexiMAP detects differential polyadenylation events in simulated data with high specificity and outperforms in sensitivity DaPars and APAtrap at small fold changes**

Percent of transcripts detected as significant (adjusted *p*-value <0.05) for every simulated fold change in the “short” region of the 3’UTR using flexiMAP, DaPars, APAtrap and Roar. Only transcripts where the polyadenylation site has been correctly predicted by DaPars and APAtrap are included in this plot. flexiMAP clearly outperformed Dapars and APAtrap for small fold changes. In addition, DaPars and APAtrap contain more false positives (see results for fold change=1). Although application of the recommended post-hoc filters (PDUI for DaPars and PD for APAtrap, in dark blue and dark red respectively) corrected the false positives problem, they did so at the cost of removing the majority of events from all remaining fold change categories. The Roar method is sensitive to the use of the filtering parameter nUnderCutOff. When set to 100% (as used in the Roar documentation) Roar is a lot less sensitive than flexiMAP and less sensitive than the other two methods for fold changes <=2. We set this filter here to 50% for a more fair comparison. This makes Roar the most sensitive method at low fold changes but at the cost of making it the least specific of all methods (highest number of false positives at fold change 1).

# Supplementary Figure 3

**Beta-regression modeling is sensitive and outperforms the GLM quasi-binomial at low expression values**

The sensitivity of all methods that identify differential APA events increases with increased transcript expression. Notably, the beta regression model outperforms the GLM/quasi-binomial model as well as all other methods except Roar at low expression. Results shown are for transcripts with fold change 1.5.

# Supplementary Figure 4

**The flexiMAP beta-regression method is more sensitive than the GLM quasi-binomial approach for smaller numbers of samples per condition**

A GLM quasi-binomial approach detects hardly any APA events (even for highly expressed transcripts) when only a very small number of samples (<=3) per condition are available (top plot), indicating that this approach is not promising for the analysis of common RNA-seq datasets. Sensitivity is much improved by applying beta-regression (bottom plot) but at the cost of a small fraction of false positives. DaPars and APAtrap results are similar; both are independent of the number of samples but require larger fold changes to display increased sensitivity. In these plots all sites have been included (i.e. no filtering has been applied to remove sites not predicted accurately by the DaPars or APAtrap alternative polyadenylation site prediction algorithms).

# Supplementary Figure 5

**FlexiMAP’s sensitivity is not affected by the length of the 3’ UTR**

The sensitivity of flexiMAP shows little dependence on the mean 3’ UTR length (results shown are for transcripts exhibiting a fold change of 1.5 in the simulated dataset).

# Supplementary Figure 6

**FlexiMAP avoids false positive calls by modelling the presence of additional known covariates**

#

# DaPars, APAtrap and Roar report a large number of false positives for an imbalanced simulated dataset where two factors, age and sex, confound the data. In contrast, flexiMAP reports two false positives in this case, highlighting its main advantage over alternative approaches.

# Supplementary Figure 7

Percent of transcripts detected as significant (adjusted *p*-value <0.05) for every simulated fold change in the “short” region of the 3’UTR applying every available function in the *betareg* R package linking beta-distributed responses with means. Differences between the different link functions are very small with the “loglog” link function performing slightly better than all others tested.

# Supplementary Figure 8

**Quality control assessment of simulated data**

A) Density plots of the lengths of 3’UTR in each of the 11 groups in the simulated dataset indicates no length biases between the groups. Each group comprises 1000 transcripts and represents a different fold change.

B) Distribution of distances between simulated and predicted (by DaPars) proximal polyadenylation sites. Where the predicted site differed from the true site by up to 25 nucleotides in either direction, the prediction was considered correct and the corresponding transcript was kept in the simulation. These cases are highlighted in brown in the plot.

C) Transcripts with correctly predicted proximal polyadenylation sites are uniformly distributed across the fold change groups in the simulated dataset.

# Supplementary Figure 9

**Schematic diagram of flexiMAP workflow**
